# Supplementary material for: Tunable control of insect pheromone biosynthesis in Nicotiana benthamiana
Source: Plant Biotechnol J. 2023 Apr 9;21(7):1440–53. doi: 10.1111/pbi.14048 (PMC10281601; doi:10.1111/pbi.14048)
Supplement: Supplementary file 1 — Figure S1 Construct architecture influences expression from constitutive promoters. [file PBI-21-1440-s005.pdf]

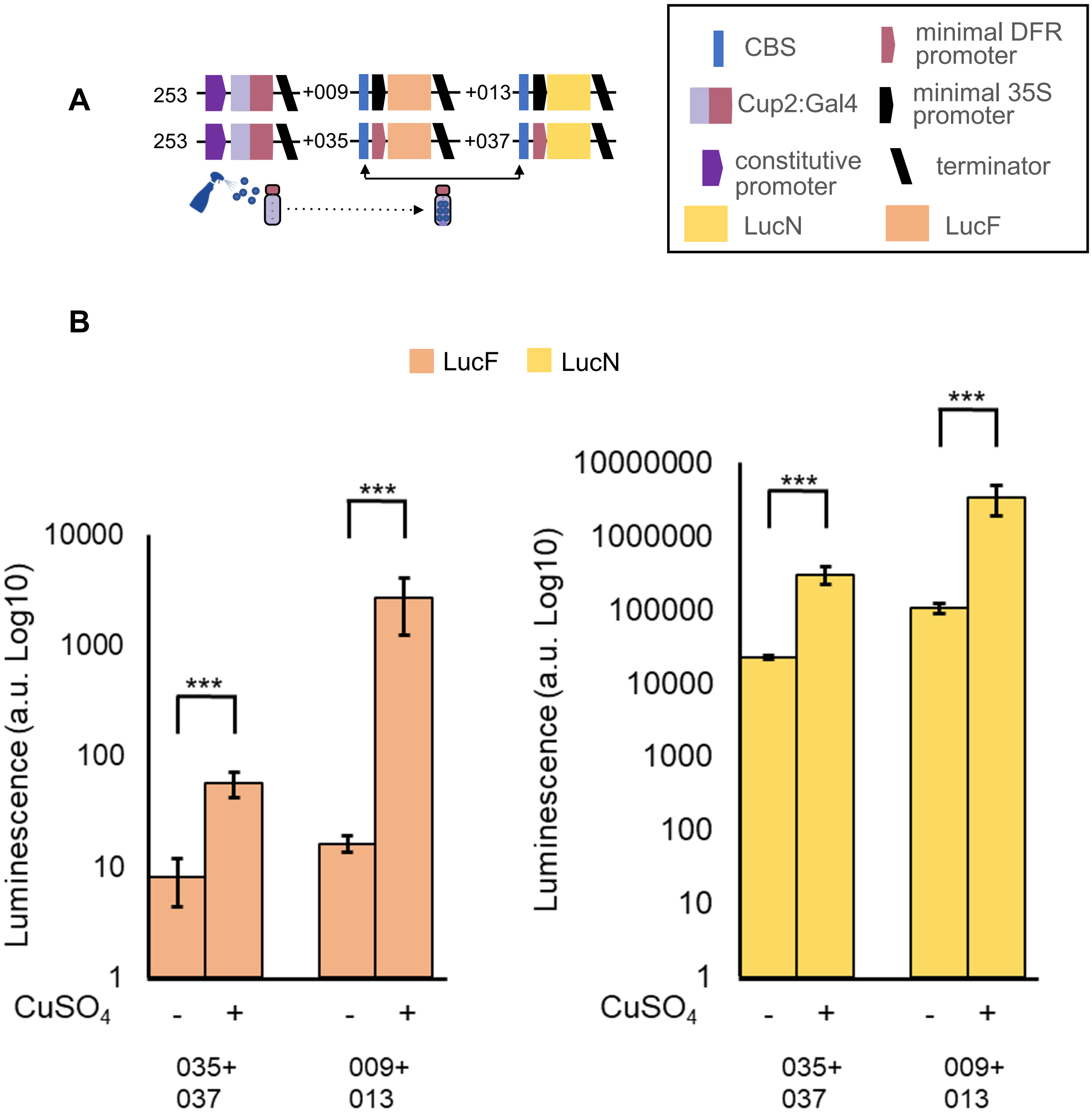

Supplementary Figure S1. Copper sulfate can induce Copper inducible promoters with minimal DFR or minimal 35S. (**A**) Schematics of plant expression constructs containing synthetic genes for copper inducible expression of firefly luciferase (LucF) and nanoluciferase (LucN). (**B**) Copper inducible promoters with minimal 35S or minimal DFR can be induced with copper sulfate (2.5 mM). Values shown are the mean and standard error of n=10 biological replicates (independent infiltrations) and differences were analyzed using pairwise Wilcoxon rank sum test with Benjamini-Hochberg correction (***$P \leq 0.001$)
