# Supplementary material for: Tunable control of insect pheromone biosynthesis in Nicotiana benthamiana
Source: Plant Biotechnol J. 2023 Apr 9;21(7):1440–53. doi: 10.1111/pbi.14048 (PMC10281601; doi:10.1111/pbi.14048)
Supplement: Supplementary file 2 — Figure S2 Copper inducible, CRISPR/Cas9‐mediated control of pheromone biosynthesis. [file PBI-21-1440-s001.pdf]

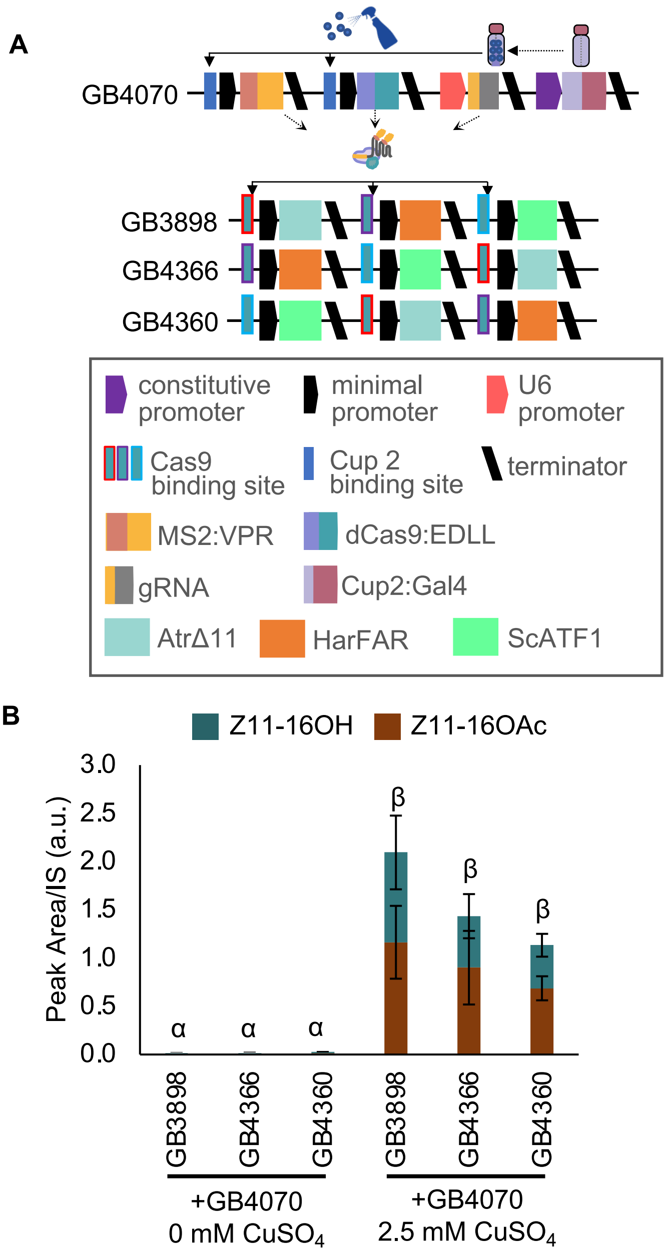

Supplementary Figure S2. Copper inducible, CRISPR/Cas9-mediated control of pheromone biosynthesis. (**A**) Schematic of plant expression constructs containing elements for copper inducible expression of the dCasEV2.1 transcriptional activator (above) and multigene constructs containing coding sequences for AtrΔ11, HarFAR and ScATF1. The latter are assembled with a promoter consisting of a minimal DFR core promoter fused to one of three unique sequences containing the conserved gRNA target sites. (**B**) Application of $CuSO_4$ results in dCasEV2.1 mediated production of the pheromone components (Z11-16OH and Z11-16OAc). Values shown are the mean and standard error of n=3 biological replicates (independent infiltrations). Means followed by a common Greek letter (α, β) are not significantly different (one-way ANOVA with post-hoc Tukey HSD at the 5% level of significance).
