# Supplementary material for: Tunable control of insect pheromone biosynthesis in Nicotiana benthamiana
Source: Plant Biotechnol J. 2023 Apr 9;21(7):1440–53. doi: 10.1111/pbi.14048 (PMC10281601; doi:10.1111/pbi.14048)
Supplement: Supplementary file 3 — Figure S3 Transcription of dCas9:EDLL and MS2:VPR in T1 CBS:dCas transgenic plants. [file PBI-21-1440-s003.pdf]

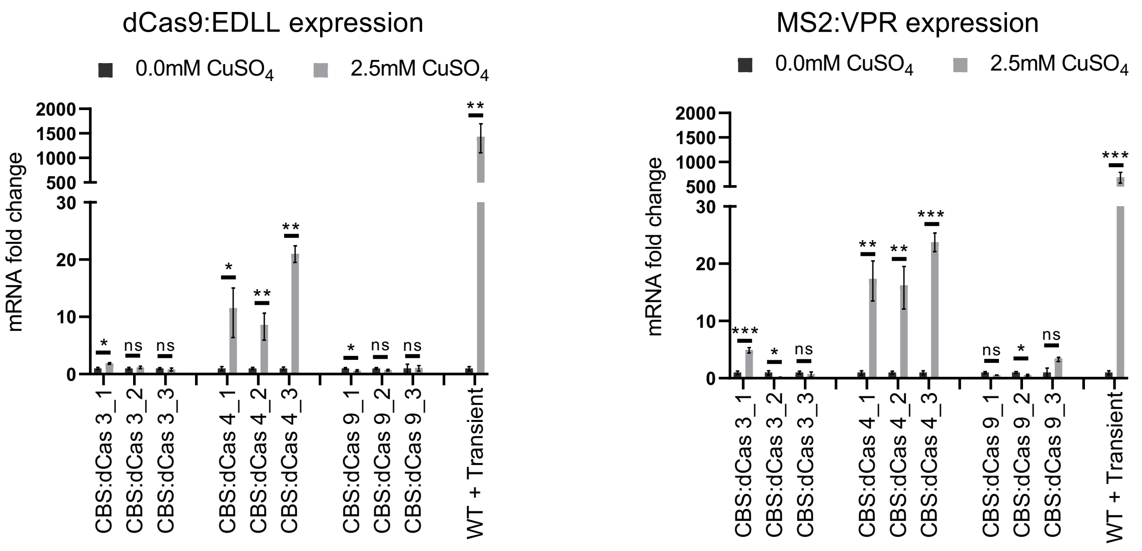

Supplementary Figure S3. Transcription of dCas9:EDLL and MS2:VPR in T_1 CBS:dCas transgenic plants. Three leaves from each of three transgenic progeny (T_1) of three independent transgenic T0 lines were selected and 0.0mM $CuSO_4$ or 2.5mM $CuSO_4$ applied each side of the midrib. Samples were collected from each plant 2 days after the induction (5dpi for the transient constructs). mRNA levels, relative to expression of the F-BOX gene (ΔCt) and and the ΔΔCt used to calculate fold change between copper concentrations. A WT plant infiltrated with the CBS:dCas module was included as a control. Error bars represents SD (n = 3). *P*-values were calculated using Student's t-test; **P* ≤ 0.05, ***P* ≤ 0.01, ****P* ≤ 0.001; ns= not significant.
