# Supplementary material for: Tunable control of insect pheromone biosynthesis in Nicotiana benthamiana
Source: Plant Biotechnol J. 2023 Apr 9;21(7):1440–53. doi: 10.1111/pbi.14048 (PMC10281601; doi:10.1111/pbi.14048)
Supplement: Supplementary file 4 — Figure S4 Pheromone biosynthesis in T0 transgenic Nicotiana benthamiana transgenics. [file PBI-21-1440-s002.pdf]

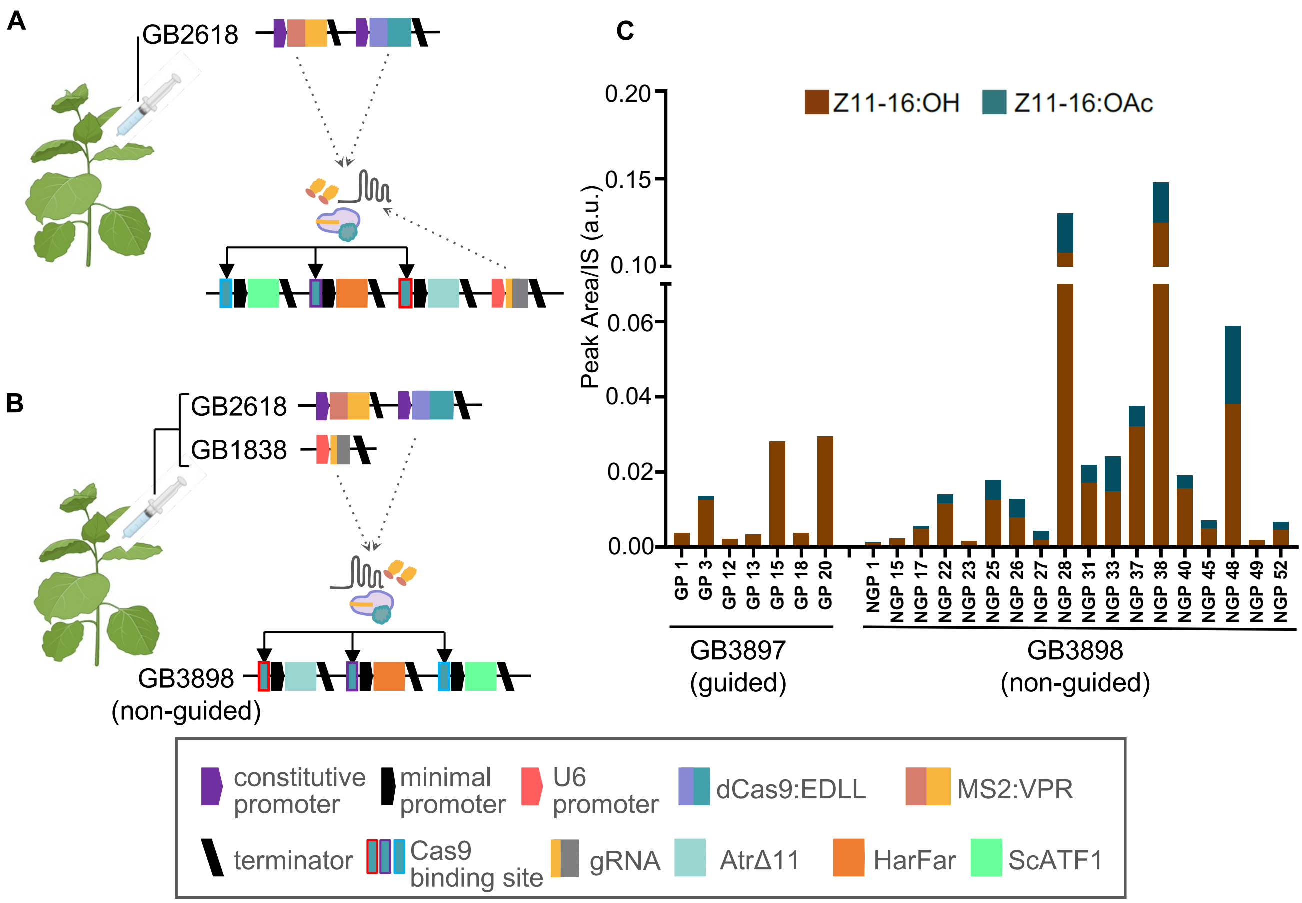

Supplementary Figure S4. Pheromone biosynthesis in T_0 transgenic *Nicotiana benthamiana* transgenics. Schematics of constructs used for expression of moth pheromones in transgenic lines containing the (**A**) guided pathway (sgRNA integrated) or (**B**) non-guided pathway (sgRNA infiltrated). (**C**) Pheromone levels obtained from T_0 transgenics infiltrated with constructs expressing regulatory elements. The figure includes images from Biorender (biorender.com).
