# Supplementary material for: Tunable control of insect pheromone biosynthesis in Nicotiana benthamiana
Source: Plant Biotechnol J. 2023 Apr 9;21(7):1440–53. doi: 10.1111/pbi.14048 (PMC10281601; doi:10.1111/pbi.14048)
Supplement: Supplementary file 5 — Table S1 Constructs used in this study. [file PBI-21-1440-s004.pdf]

Supplementary Table S1 Constructs used in this study

Plasmid Code	Contents	Addgene Code and Reference	Description
GB1203	35s:P19:nos	#68214 (Sarrion-Perdigones et al., Plant Physiol. 2013 Jul;162(3):1618-31)	Transcriptional unit for constitutive expression of the silencing suppressor P19 driven by the 35s promoter.
253 (pEPKKα1KN0253)	AtuNos+TMVΩ:CUP2:GAl4:35s	#187560 (this study)	Transcriptional unit for constitutive expression of CUP2:GAL4 driven by the nos promoter.
021 (pEPCTΩSP0021)	CBS:LucF (forward):35s + CBS:LucN (forward)	#187561 (this study)	Module for copper-inducible expression of firefly luciferase and nanoluc luciferase driven by minimal synthetic promoters with binding sites for CUP2.
022 (pEPCTΩSP0022)	CBS:LucN (forward):35s + CBS:LucF:35s (forward)	#187562 (this study)	Module for copper-inducible expression of nanoluc luciferase and firefly luciferase driven by minimal synthetic promoters with binding sites for CUP2.
009 (pEPCTαKN009)	CBS:LucF:35s	#187563 (this study)	Transcriptional unit for copper-inducible expression of firefly luciferase driven by a minimal synthetic promoter with binding sites for CUP2.
013 (pEPCTαKN013)	CBS:LucN (forward):35s	#187564 (this study)	Transcriptional unit for copper-inducible expression of nanoluc luciferase driven by a minimal synthetic promoter with binding sites for CUP2.
017 (pEPCTΩSP0017)	35s:LucF (forward):35s + 35s:LucN (forward)	#187565 (this study)	Module for constitutive expression of firefly luciferase and nanoluc luciferase driven by 35s promoters.
018 (pEPCTΩSP0018)	35s:LucN (forward):35s + 35s:LucF (forward)	#187566 (this study)	Module for constitutive expression of nanoluc luciferase and firefly luciferase driven by 35s promoters.
019 (pEPCTΩSP0019)	35s:LucF (forward):35s + 35s:LucN (reverse)	#187567 (this study)	Module for constitutive expression of firefly luciferase and nanoluc luciferase driven by 35s promoters.
001 (pEPCTαKN001)	35S:LucF:35S	#187568 (this study)	Transcriptional unit for constitutive expression of firefly luciferase driven by the 35s promoter.
005 (pEPCTαKN005)	35S:LucN:35S	#187569 (this study)	Transcriptional unit for constitutive expression of nanoluc luciferase driven by the 35s promoter.
GB UA 114 A	35S:Gal4:PhiC31:35S	#187570 (Vazquez-Vilar et al., Nucleic acids research vol. 45,4 (2017): 2196-2209)	Transcriptional unit for constitutive expression of Gal4:ΦC31 driven by the 35s promoter.

Plasmid Code	Contents	Addgene Code and Reference	Description
pEPKKα2KN0100	2xOpattB-min 35S:LucN:g7	#154621 (Cai *et al.*, Nucleic Acids Res. 2020 Dec 2;48(21): 11845-11856)	Transcriptional unit for Gal4:**Φ**C31-activated expression of nanoluciferase.
pEPKKα2KN0101	4xOpattBt-min 35S:LucN:g7	#154622 (Cai *et al.*, Nucleic Acids Res. 2020 Dec 2;48(21): 11845-11856)	Transcriptional unit for Gal4:**Φ**C31-activated expression of nanoluciferase.
pEPKKα2KN0102	6xOpattBt-min 35S:LucN:g7	#154623 (Cai *et al.*, Nucleic Acids Res. 2020 Dec 2;48(21): 11845-11856)	Transcriptional unit for Gal4:**Φ**C31-activated expression of nanoluciferase.
pEPKKα1RKN0115	AtuNos:TALE:35S	#187571 (Cai *et al.*, Nucleic Acids Res. 2020 Dec 2;48(21): 11845-11856)	Transcriptional unit for constitutive expression of a TALE driven by the nos promoter.
pEPKKα2KN0091	1xTALEbs-min 35S:LucNc:g7	#154618 (Cai *et al.,* Nucleic Acids Res. 2020 Dec 2;48(21): 11845-11856)	Transcriptional unit for TALE-activated expression of nanoluciferase.
pEPKKα2KN0092	2xTALEbs-min 35S:LucNc:g7	#154619 (Cai *et al.*, Nucleic Acids Res. 2020 Dec 2;48(21): 11845-11856)	Transcriptional unit for TALE-activated expression of nanoluciferase.
pEPKKα2KN0093	4xTALEbs-min 35S:LucNc:g7	#154620 (Cai *et al.*, Nucleic Acids Res. 2020 Dec 2;48(21): 11845-11856)	Transcriptional unit for TALE-activated expression of nanoluciferase.
GB2085	35s:Ms2VPR:nos + 35s:dCas9:EDLL:nos	#160645 (Selma *et al.*, Plant Biotechnol J. 2019 Sep;17(9):1703)	Module for the expression of dCas9 fused to EDLL and Ms2 protein fused to VPR.
GB1724	U626:gRNA4(pNOS)	#160621 (Selma *et al.,* Plant Biotechnol J. 2019 Sep;17(9):1703)	Transcriptional unit for a gRNA targeting the nos promoter with a MS2 recognition loop.
GB1838	U6-26-1gRNA(pDFR)	#160625 (Selma *et al.*, Plant Biotechnol J. 2019 Sep;17(9):1703)	Transcriptional unit for the expression of a g RNA targeting the DFR promoter with two copies of the MS2 aptamer.
GB2513	35s:dCas9:EDLL:nos +35s:MS2:VPR:nos + U626:gRNA1 (pDFR)	#187803 (this study)	Module for constitutive expression of dCas9:EDLL, Ms2:VPR and a gRNA targeting the DFR promoter.
GB1024	35s:AtrΔ11:35s + 35s:HarFAR:35s	#187804 (this study)	Module for the constitutive expression of the Δ11 desaturase from *Amyelois transitella* and a fatty acid reductase from *Helicoverpa armigera*.
GB1022	35s:EaDAct:35s	#187805 (this study)	Transcriptional unit for expression of diacylglycerol acetyltransferase from *Euonymus alatus.*

Plasmid Code	Contents	Addgene Code and Reference	Description
GB3681	35s:ScATF1:35s	#187806 (this study)	Transcriptional unit for expression of alcohol O-acetyltransferase from *Saccharomyces cerevisiae* S288C, codon optimized for Nicotiana.
GB3682	35s:SpATF1-2:35s	#187807 (this study)	Transcriptional unit for expression of alcohol O-acetyltransferase from *Saccharomyces pastorianus* strain CBS 1483 chromosome SeVIII-SeXV, codon optimized for Nicotiana.
GB3683	35s:EfDAct:35s	#187808 (this study)	Transcriptional unit for expression of 1,2-diacyl-sn-glycerol:acetyl-CoA acetyltransferase from *Euonymus fortunei*, codon optimized for Nicotiana.
678 (pEPKKΩ1SP0678)	35s:TMVΩ:CUP2:GAl4: nos + CBSmin35s:AAtrΔ11:35 s + CBSmin35s: ATF1-2:mas + CBSmin35s:HarFAR:g7	#187605 (this study)	Module for copper inducible expression of AtrD11, HarFAR and EaDAct.
GB3897	minDFR:ATF1:mtb + minDFRHarFAR:pds + minDFR:AtrΔ11:dfr+ U626:gRNA1 (pDFR)	#187809 (this study)	Module for dCasEV2.1 activated expression of AtrD11, HarFAR and ScATF1 plus gRNA-1DFR.
GB4068	nos:CUP2:GAL4:nos + CBS:dCas9:EDLL:nos + CBS: MS2:VPR;nos	#187810 (this study)	Module for the constitutive expression of Cup2:Gal4AD and copper-inducible expression of dCasEV2.1 (dCas9:EDLL and MS2:VPR).
GB4070	nos:CUP2:GAl4:nos + U626:gRNA (DFR) + CBS:dCas9:EDLL:nos + CBS:MS2:VPR:nos	#187811 (this study)	Module for the constitutive expression of Cup2:Gal4AD and gRNA-1 DFR, and the copper-inducible expression of dCasEV2.1 (dCas9:EDLL and MS2:VPR).
GB2815	pUPD2_GB_SynP (A1) Random Sequence R1	#193112 (Moreno-Giménez *et al.*, ACS Synth. Biol. 2022, 11, 9, 3037–3048)	Random sequence R1 of 1240 bp for A1 distal promoter position.
GB3269	pUPD2_GB_SynP (A1) Random Sequence R2	#193113 (Moreno-Giménez *et al.*, ACS Synth. Biol. 2022, 11, 9, 3037–3048)	Random sequence R2 of 1240 bp for A1 distal promoter position.
GB3270	pUPD2_GB_SynP (A1) Random Sequence R3	#193114 (Moreno-Giménez *et al.*, ACS Synth. Biol. 2022, 11, 9, 3037–3048)	Random sequence R3 of 1240 bp for A1 distal promoter position.
GB3275	pUPD2_GB_SynP (A2) G1abc.2	#193129 (Moreno-Giménez *et al.*, ACS Synth. Biol. 2022, 11, 9, 3037–	A2 Proximal promoter sequence consisting of three times the target sequence for the gRNA-1 DFR (gRNA1) flanked by random sequences.

		3048)	
GB3276	pUPD2_GB_SynP (A2) G1abc.3	#193130 (Moreno-Giménez *et al.*, ACS Synth. Biol. 2022, 11, 9, 3037–3048)	A2 Proximal promoter sequence consisting of three times the target sequence for the gRNA-1 DFR (gRNA1) flanked by random sequences.
GB3277	pUPD2_GB_SynP (A2) G1abc.4	#193131 (Moreno-Giménez *et al.*, ACS Synth. Biol. 2022, 11, 9, 3037–3048)	A2 Proximal promoter sequence consisting of three times the target sequence for the gRNA-1 DFR (gRNA1) flanked by random sequences.
GB2566	pUPD2_miniDFR	#193104 (Moreno-Giménez *et al.*, ACS Synth. Biol. 2022, 11, 9, 3037–3048)	Minimal promoter of SlDFR gene containing 62bp upstream the transcription start site and the 5'UTR region.
GB3898	minDFR:AtrΔ11:dfr + minDFR:HarFAR:pds + minDFR:ScATF1:mtb	#187812 (this study)	Module for dCasEV2.1 activated expression of AtrΔ11, HarFAR and ScATF1.
GB4356	minDFR:AtrΔ11:dfr + minDFR:HarFAR:pds + minDFR:SpATF1-2:mtb	#187813 (this study)	Module for dCasEV2.1 activated expression of AtrΔ11, HarFAR and SpATF1-2.
GB4360	minDFR:ScATF1:mtb + minDFR:AtrΔ11:dfr + minDFR:HarFAR	#187814 (this study)	Module for dCasEV2.1 activated expression of ATF1, AtrΔ11 and HarFAR.
GB4361	minDFR:SpATF1-2:mtb + minDFR:AtrΔ11:dfr + minDFR:HarFAR:pds	#187815 (this study)	Module for dCasEV2.1 activated expression of ATF1-2, HarFAR and AtrΔ11.
GB4366	minDFR:HarFAR:pds + minDFR:ScATF1:mtb + minDFR:AtrΔ11:dfr	#187816 (this study)	Module for dCasEV2.1 activated expression of HarFAR, ATF1 and AtrΔ11.
GB4367	minDFR:HarFAR:pds + minDFR:SpATF1-2:mtb + minDFR:AtrΔ11:dfr	#187817 (this study)	Module for dCasEV2.1 activated expression of HarFAR, ATF1-2 and AtrΔ11.
